# A balancing act: RNA binding protein HuR/TTP axis in endometriosis patients

**DOI:** 10.1038/s41598-017-06081-7

**Published:** 2017-07-19

**Authors:** Kasra Khalaj, Soo Hyun Ahn, Mallikarjun Bidarimath, Yasmin Nasirzadeh, Sukhbir S. Singh, Asgerally T. Fazleabas, Steven L. Young, Bruce A. Lessey, Madhuri Koti, Chandrakant Tayade

**Affiliations:** 1 Department of Biomedical and Molecular Sciences, Queen’s University, Kingston, Ontario, K7L 3N6 Canada; 2 Department of Obstetrics and Gynecology, University of Ottawa, Ottawa, Ontario, K1H 7W9 Canada; 3 Department of Obstetrics, Gynecology and Reproductive Biology, Michigan State University College of Human Medicine, Grand Rapids, MI 49503 USA; 4 Department of Obstetrics and Gynecology, University of North Carolina, Chapel Hill, North Carolina, NC 27514 USA; 5 Department of Obstetrics and Gynecology, Greenville Health Systems, Greenville, South Carolina, SC 29605 USA

## Abstract

Endometriosis, a major reproductive pathology affecting 8–10% of women is characterized by chronic inflammation and immune dysfunction. Human antigen R (HuR) and Tristetraprolin (TTP) are RNA binding proteins that competitively bind to cytokines involved in inflammation including: tumor necrosis factor alpha (TNF-α), granulocyte macrophage colony stimulating factor (GM-CSF), interleukin 6 (IL-6) among others, and stabilize and destabilize them, respectively. The aim of this study was to examine RNA binding protein (RNABP) HuR/TTP axis in endometriosis patients compared to menstrual stage matched healthy fertile controls in hopes of better understanding their contribution to the pathogenesis of endometriosis. Additionally, using a targeted *in vitro* siRNA approach, we examined whether knock-down of TTP can play a functional role on other RNABPs that competitively bind to inflammatory targets of TTP in both endometriotic and endometrial epithelial cell lines. Our results suggest that RNABPs TTP and HuR are dysregulated in endometriotic lesions compared to matched eutopic patient samples as well endometrium from healthy controls. Silencing of TTP in endometriotic and endometrial epithelial cells revealed differential response to inflammatory cytokines and other RNABPs. Our results suggest potential involvement of HuR/TTP RNA binding protein axis in regulation of inflammation in endometriosis.

## Introduction

Endometriosis is a reproductive pathology characterized by growth of endometrium-like tissue outside of the uterus in ectopic sites^1, 2^. It is now well established that chronic inflammatory *milieu* in the peritoneal cavity of endometriosis patients contributes to pain and infertility associated with the disease^3^. Despite decades of research, current treatment option transiently alleviate but do not cure the disease. The most widely accepted Sampson’s theory of retrograde menstruation, postulates that endometrial fragments from retrograde menstruation lead to endometriosis pathogenesis^4^. This theory however remains to adequately address why although 78–90% of women experience retrograde menstruation, endometriosis only affects 8–10% of all women of reproductive age, irrespective of ethnicity^2, 5–7^. Previous research has shown strong evidence of increased pro-inflammatory and decreased anti-inflammatory cytokines in the peritoneal fluid in endometriosis patients^8^. Indeed, we have shown endometriotic lesions are primary drivers of inflammation and surgical removal of lesions leads to temporary decline in the levels of pro-inflammatory cytokines in systemic circulation of endometriosis patients^9^. We have also shown that endometriotic lesions and eutopic endometrium of endometriosis patients have unique immune-inflammation gene signatures^9^. Other work from our lab has shown that IL-17A can contribute to lesion development via both promotion of inflammation and stimulating production of angiogenic cytokines^10^. This evidence clearly suggests that inflammation is central to the progression of endometriosis associated pain and potential infertility. However, it is not clear whether inflammation contributes to the pathogenesis of endometriosis or is a by-product of endometriotic lesion establishment. It is also not clear whether and how inflammation is regulated in endometriosis.

Tristetraprolin (TTP) is an RNA binding protein (RNABP) encoded by ZFP36 in humans (Zfp36 in mice) that can bind and destabilize mRNA transcripts of important inflammatory or inflammation-related cytokines including: tumor necrosis factor alpha (TNF-α)^11^, granulocyte macrophage colony stimulating factor (GM-CSF)^12^, cyclooxygenase-2 (COX-2)^13^, Interleukins IL-1a, IL-2, IL-3, IL-6, IL-10, IL-12 and IL-23^14–18^. Tristetraprolin exerts its destabilizing function via binding to AU-rich element (ARE) regions that reside within the 3′ untranslated region (UTR) of a variety of mRNA transcripts, which ultimately leads to degradation of the target^19^. The TTP or TIS11 family of RNA binding proteins have been previously characterized in normal human tissues and all three members were found to be expressed in moderate to high levels in both the ovary and cervix, and thus are expressed in physiologically relevant levels throughout the female reproductive tract, including the placenta^20^. Recently, TTP has been identified as a global post-transcriptional regulator of inflammation, signifying its conserved importance in maintenance of homeostatic balances of cytokine production^21^.

Human antigen R (HuR), or *ELAVL1* is another RNA-binding protein expressed within the reproductive tract in humans. This RNA-binding protein has similar functions to TTP; to bind to inflammatory cytokines, however its role is opposite to that of TTP, by stabilizing the transcripts of the same cytokines^22^. HuR thus acts as an RNA-stabilizer, while TTP acts as a RNA-destabilizing factor. Human antigen R has been previously reported to be important for early placental and embryonic development^23^. Previous work from our group has shown that all three members of the TTP gene family are expressed and are biologically relevant at the maternal-interface in a fetal loss model in pigs^24^. Our group has also shown relevance of RNABPs including TTP and HuR as well as localization of TTP in inflammatory conditions using a mouse model of recurrent pregnancy loss^25^. Other RNA binding proteins such as AUF1 have also been shown to have indirect effects by influencing methylation under hypoxic conditions in endometriosis^26^. The adult stem cell marker RNA binding protein, Musashi-1 has been shown to be increased in endometriosis and endometrial carcinomas^27^. Unlike Musashi-1, an RNA binding protein involved in programmed cell death, TIA-1 cytotoxic granule-associated RNA binding protein, is downregulated in the ectopic endometrium. Additionally, previous work with HuR has shown differential expression in ectopic endometriotic lesions compared to eutopic endometrium and that HuR expression varies during the menstrual cycle^28^. However, the mechanisms that might regulate this difference in expression pattern and hormonal effects have yet to be determined.

In this study, we sought to understand whether HuR and TTP family members regulate the inflammatory response during endometriotic lesion formation and development. We hypothesized that the HuR/TTP axis modulates the inflammatory response at both the site of the endometriotic lesions as well as the local peritoneal microenvironment via aberrant expression or dysregulation of these regulators. Our main objectives were to determine the *in vivo* and *in vitro* associations of TTP and HuR and their selected pro-inflammatory cytokine targets in matched ectopic and eutopic samples from patients and in healthy fertile controls. Our other objective was to characterize the TTP family using a syngeneic mouse model of endometriosis. We also sought to determine the mechanisms of HuR/TTP function via *in vitro* RNA interference/silencing in endometriotic epithelial (12Z) and endometrial epithelial carcinoma (EECCs) cells.

## Results

### ZFP36, ZFP36L1, ZFP36L2 and ELAVL1 mRNA is expressed in endometriosis patient samples

**Figure 1:**
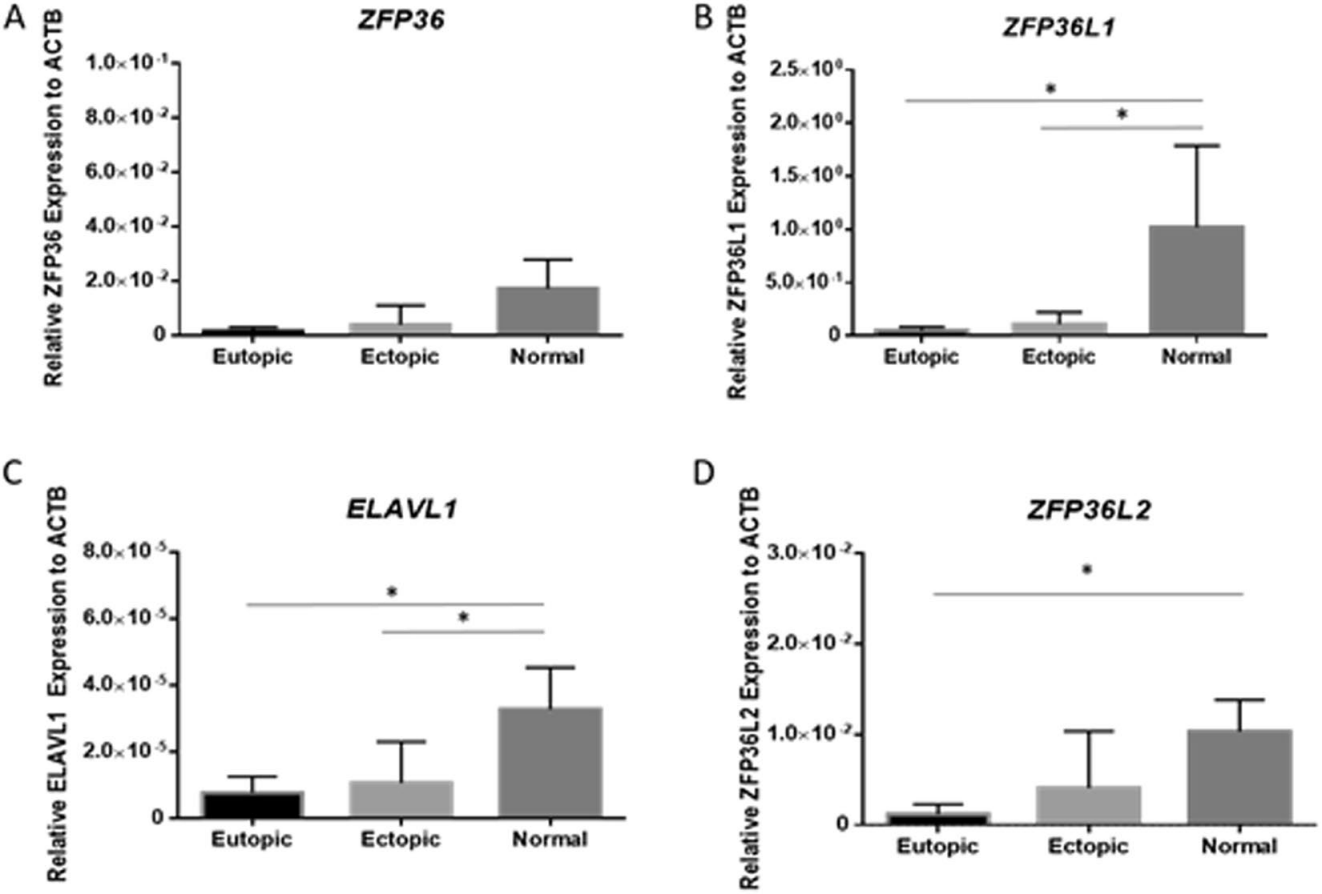
HuR/TTP axis mRNA levels are altered in patients with endometriosis: RNA binding protein expression profiles in endometriosis patients and in normal fertile controls. mRNA transcripts for *ZFP36* remain unchanged (Panel A). Transcripts for *ZFP36L1, ELAVL1* were significantly downregulated in eutopic and ectopic endometrium from endometriosis patients when compared to normal endometrium from fertile healthy controls (Panels B,C). Transcripts for *ZFP36L2* was significantly downregulated in eutopic endometrium from patients with endometriosis compared to fertile healthy control women (Panel D). Expression data illustrated as mean ± SEM. *P < 0.05. Sample groups consisted of n = 22 human eutopic endometrium, n = 22 human ectopic endometriotic lesions, and n = 16 normal control endometrium.

The mRNA of *ZFP36* (encoding TTP), *ZFP36L1* (encoding Tis11b), *ZFP36L2* (encoding Tis11d) and *ELAVL1* (encoding HuR) were expressed in matched ectopic lesion and eutopic endometrium from endometriosis patients, as well as in endometrial samples from menstrual stage matched fertile subjects with no evidence of endometriosis. The mRNA of *ZFP36L1* in eutopic endometrium and ectopic endometriotic lesions was significantly lower than in endometrial tissue from controls (Fig. 1 Panel B; p < 0.05). The same was also observed with *ELAVL1* (Fig. 1 Panel C; p < 0.05). Significantly lower mRNA of *ZFP36L2* in eutopic endometrium from patients was observed compared to normal healthy endometrium (Fig. 1 Panel D; p < 0.05).

### HuR and TTP protein is differentially expressed in endometriosis patients compared to endometrial biopsies from healthy controls

**Figure 2:**
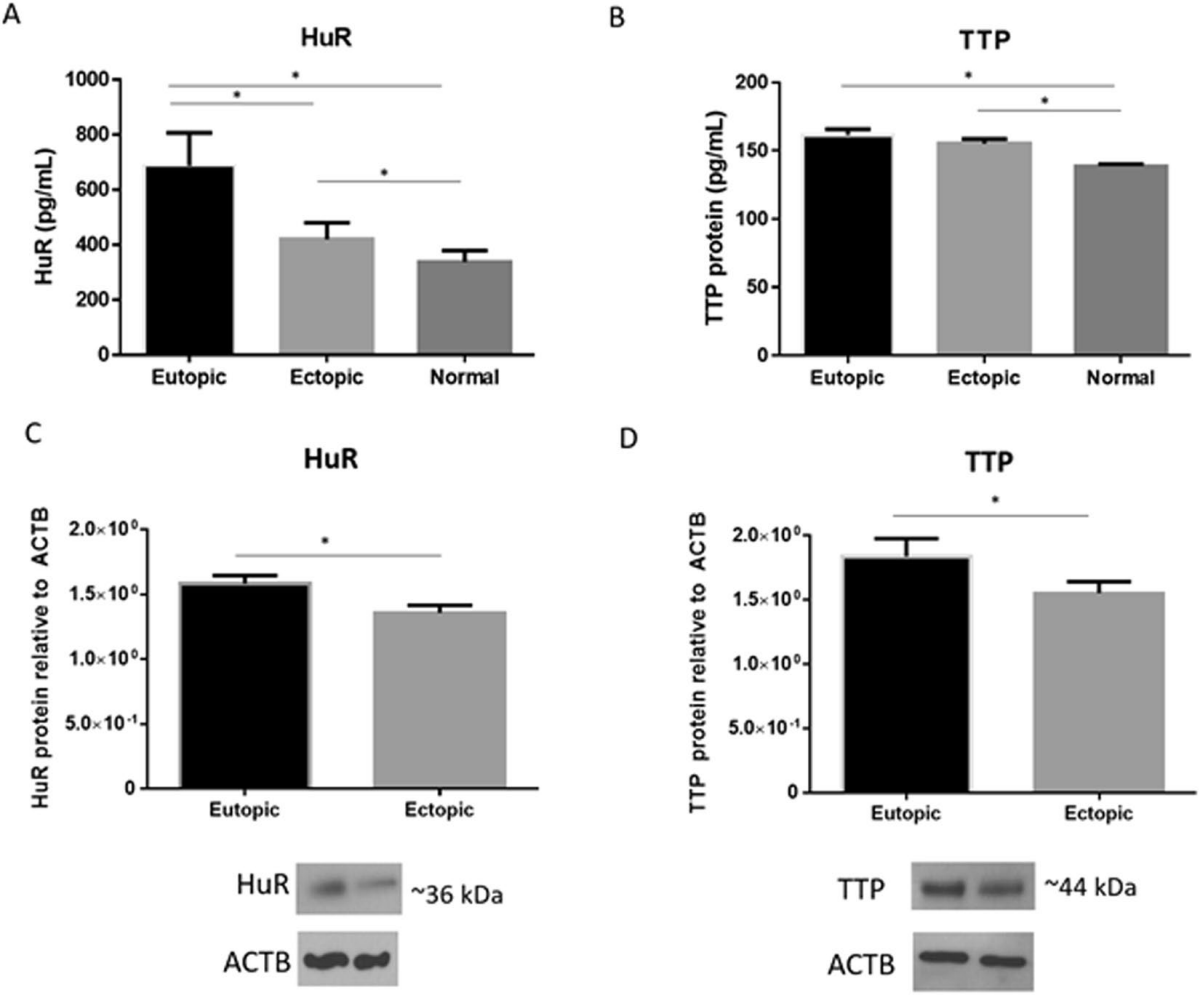
HuR/TTP axis protein levels are altered in patients with endometriosis: characterization using ELISA and western blotting in endometriosis patients and in normal fertile controls. Expression of HuR was significantly higher in eutopic endometrium compared to matched ectopic lesions from endometriosis patients as well as normal endometrium from healthy controls (Panel A). TTP protein was significantly higher in eutopic and ectopic matched endometrium from endometriosis patients compared to normal endometrium from healthy controls (Panel B). In a different cohort of women with endometriosis receiving dienogest, HuR and TTP protein was significantly higher in matched eutopic endometrium compared to ectopic lesions (Panels C,D, densitometry as ratio to ACTB and cropped blots are displayed. Full blot images are shown in Supplemental Figs 1 and 2). Expression data illustrated as mean ± SEM. *P < 0.05. Panels A,B (Greenville patient subset) consisted of n = 22 human eutopic endometrium, n = 22 human ectopic endometriotic lesions, n = 16 normal control endometrium. Panels C,D (Ottawa patient subset) consisted of n = 8 human eutopic endometrium and n = 8 human ectopic endometriotic lesions.

Human antigen R and TTP protein expression were evaluated in endometriosis patients and control subjects using enzyme linked immunosorbent assay (ELISA) as well as western blotting. Significantly higher HuR protein was detected in both matched ectopic and eutopic endometrium from patients compared to normal control endometrium from women with no evidence of endometriosis (Fig. 2 Panel A; p < 0.05). Additionally, significantly higher HuR protein was detected in eutopic endometrium compared to ectopic endometriotic tissues from patients (Fig. 2 Panel A; p < 0.05). Expression of TTP was similar to HuR, however to a much lesser extent. TTP was significantly downregulated in normal endometrium compared to ectopic and eutopic endometrium from endometriosis patients (Fig. 2 Panel B; p < 0.05). In order to further support our ELISA findings, western blots were performed in a different cohort of patients with endometriosis (The Ottawa Hospital, Ottawa, Canada), for both HuR and TTP. Bands were detected in both eutopic and ectopic endometrium at 36 kDa for HuR and 44 kDa for TTP, matching the predicted molecular weight for each protein (Fig. 2 Panels C,D). Significantly higher amounts of HuR and TTP in eutopic endometrium compared to matched ectopic endometriotic tissue was also detected using western blotting (Fig. 2 Panel C & D; p < 0.01 & p < 0.05, respectively). Overall, less TTP protein was detected in endometrium (in both patients and controls) compared to HuR (TTP; approximately 100–180 pg/ml, HuR; 250–700 pg/ml).

### Distinct profile of inflammatory cytokine targets in endometriosis patients compared to controls

**Figure 3:**
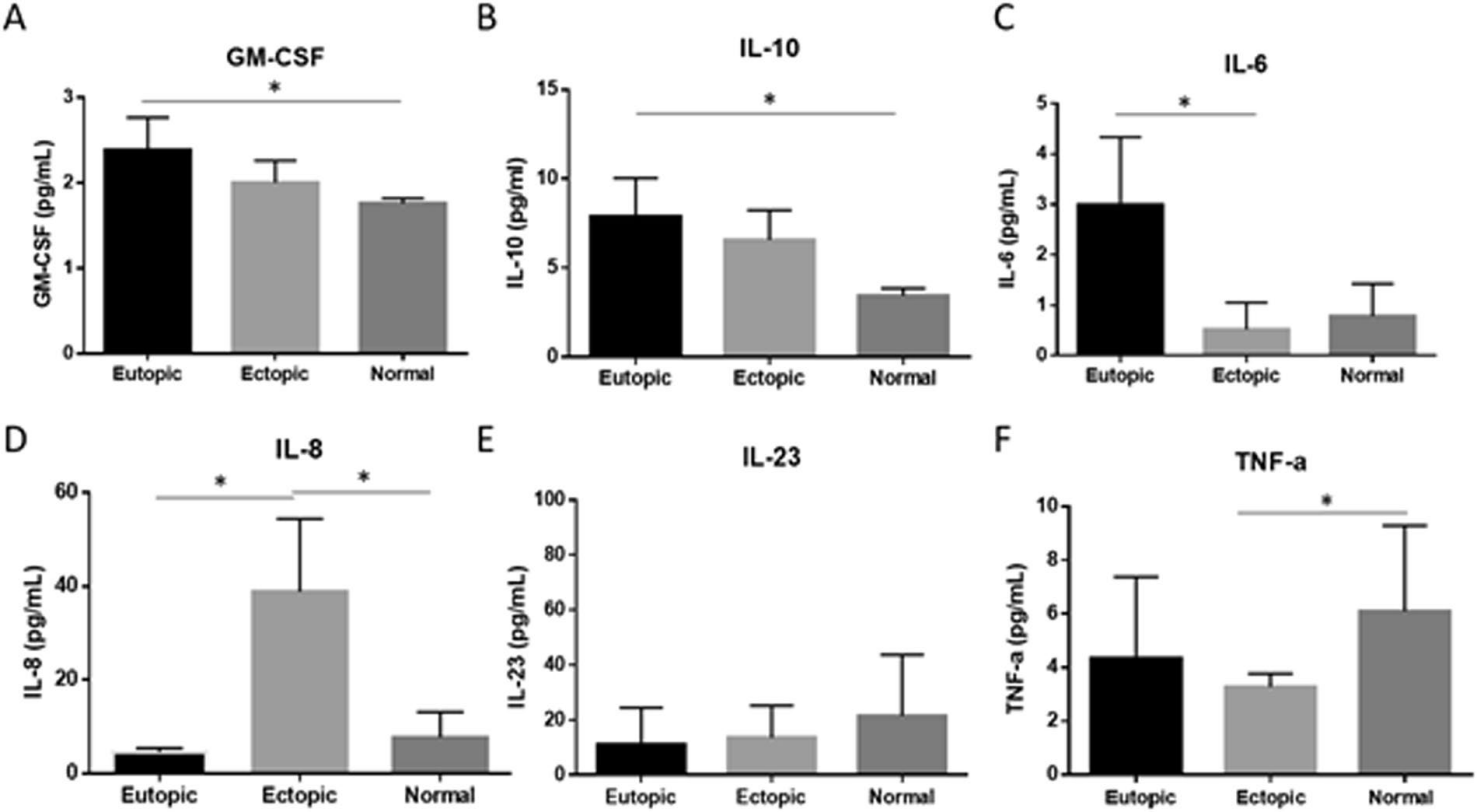
Inflammatory cytokine protein profiles in endometriosis patients and compared to normal fertile controls. Protein expression using multiplexing reveals higher GM-CSF and IL-10 in eutopic endometrium from patients compared to normal endometrium from fertile healthy controls (Panels A,B). Interleukin-6 was significantly upregulated in matched eutopic compared to ectopic lesions from patients (Panel C). Similarly, Interleukin-8 was upregulated in ectopic endometrium compared to matched eutopic endometrium from patients as well as in normal endometrium from healthy fertile controls (Panel D). Tumor necrosis factor alpha was higher in normal endometrium from healthy fertile controls compared to ectopic endometrium from endometriosis patients (Panel F). Expression data illustrated as mean ± SEM. *P < 0.05. Sample groups consisted of n = 22 human eutopic endometrium, n = 22 human ectopic endometriotic lesions, and n = 16 normal control endometrium.

Of 12 cytokines assessed (GM-CSF, IFNγ, IL-1β, IL-4, IL-5, IL-6, IL-8, IL-10, IL-12p70, IL-17, IL-23, TNF-α), 5 cytokines involved in inflammation were found to be significantly different in patients compared to controls. Significantly higher GM-CSF protein was detected in eutopic compared to matched ectopic endometrium in endometriosis patients (Fig. 3 Panel A; p < 0.05). Additionally, significantly higher GM-CSF was detected in eutopic endometrium from patients with endometriosis compared to endometrium from normal fertile controls (Fig. 3 Panel A; p < 0.05). Whereas Interleukin-10 was significantly higher in eutopic endometrium compared to normal fertile controls (Fig. 3 Panel B; p < 0.05). Interleukin-6 was significantly elevated in eutopic endometrium compared to ectopic endometriotic lesions from patients (Fig. 3 Panel C; p < 0.05). Interleukin-8 was significantly elevated in ectopic endometriotic lesions compared to both its matched eutopic endometrium as well as in normal fertile endometrium (Fig. 3 Panel D; p < 0.05). Additionally, IL-23 protein had tendency to be lower in eutopic endometrium from patients with endometriosis compared to normal fertile controls (Fig. 3 Panel E; p = 0.098). TTP’s major target, TNF-α was significantly lower in ectopic endometriotic lesions compared to normal fertile endometrium (Fig. 3 Panel F; p < 0.05).

### *Zfp36, Zfp36l1, Zfp36l2 and Elavl1* mRNA is expressed in both ectopic and eutopic endometrium in a mouse model of endometriosis

**Figure 4:**
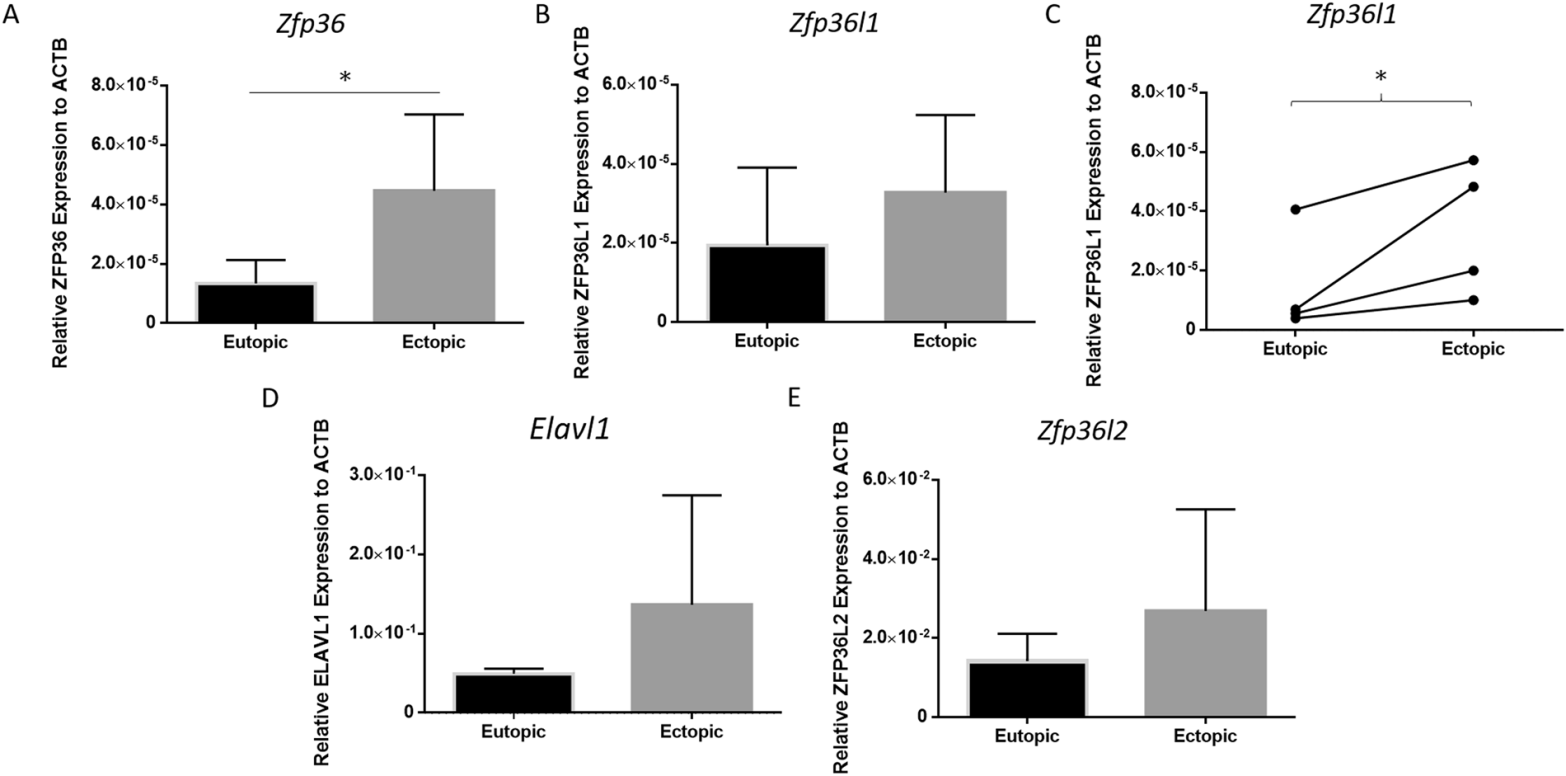
Upregulation of HuR/TTP axisRNA binding proteins *Zfp36, Zfp36l1, Zfp36l2* (TTP family), and *Elavl1* (HuR) mRNA in an *in vivo* mouse model of endometriosis. mRNA transcripts encoding for TTP (*Zfp36*) is elevated in ectopic compared to eutopic endometrium obtained from Balb/c mice (panel A). *Zfp36L1* was elevated in ectopic compared to eutopic endometrium using matched t-test (panel C), however did not significantly differ by unpaired student’s t-test (panel B). mRNA expression for *Elavl1* and *Zfp36l2* was not statistically significant (Panel D,E; p = 0.09), and did not differ by paired/matching tests. Endometriosis was induced in BALB/cByJ mice by surgical implantation of endometrium isolated from donor mice (n = 2) into the left peritoneal cavity of recipient mice. Data presented consists of Days 7 (n = 3) and 17 (n = 3) of eutopic endometrium and ectopic lesion harvest. Expression patterns did not change by collection day, however data is presented as both time points pooled n = 6. Expression data illustrated as mean ± SEM. *P < 0.05.

In order to examine if HuR/TTP axis contributes to the pathophysiology of endometriosis, we evaluated expression of *Zfp36, Zfp36l1, Zfp36l2* and *Elavl1* in eutopic and ectopic lesions obtained from syngeneic BALB/cByJ mouse model of endometriosis. The RNABP *Zfp36* was significantly upregulated in ectopic lesions compared to matched eutopic controls (Fig. 4A; p < 0.05). This mRNA difference was also observed in *Elavl1* (Fig. 4D; p < 0.05), as well as *Zfp36l1* (matched test only, Fig. 4C; p < 0.05), but not with *Zfp36l2* which encodes for Tis11d (Fig. 4E). Dysregulation of HuR/TTP axis in mouse model supports some of the findings from our endometriosis patient cohorts.

### RNABPs are differentially regulated in endometriotic epithelial 12Z cells compared to EECCs after silencing of TTP

**Figure 5:**
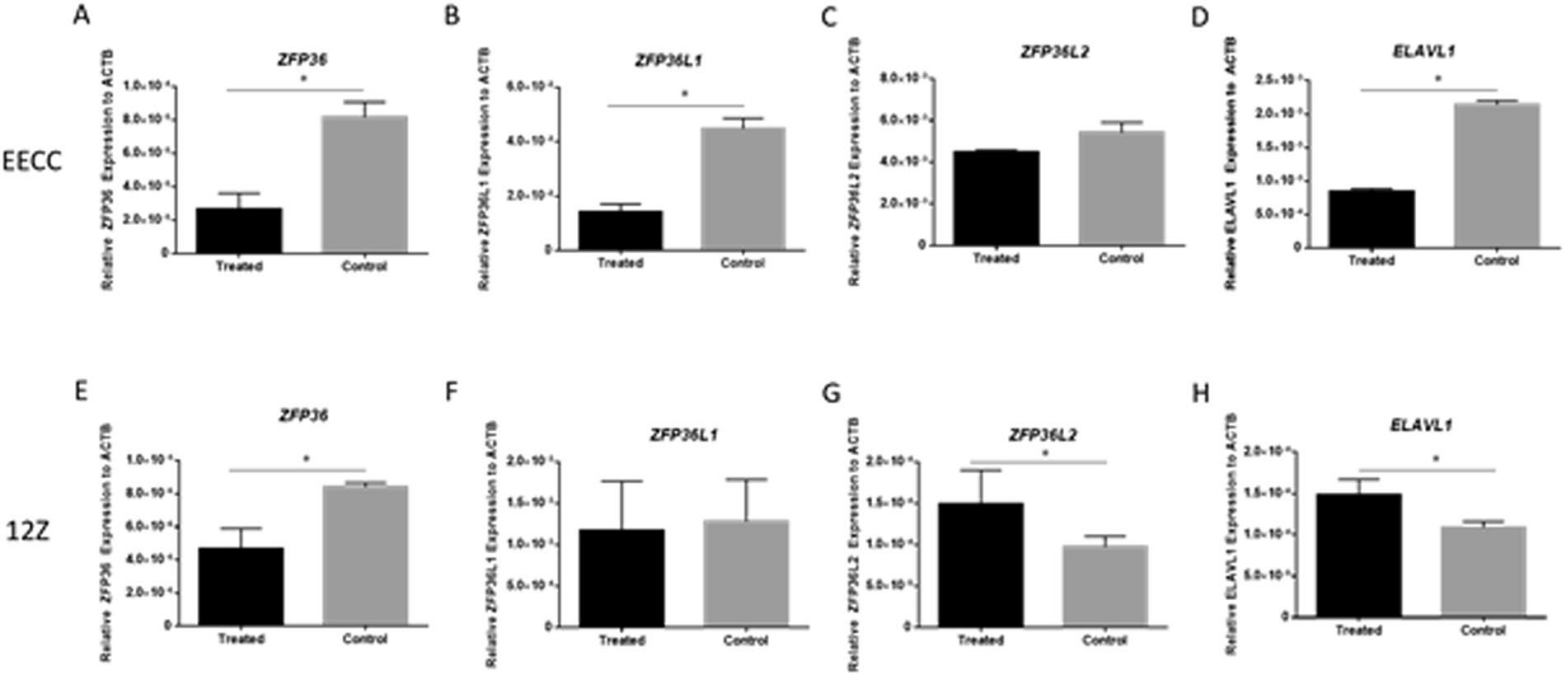
TTP knockdown results in endometriotic (12Z) and endometrial epithelial (EECC)-specific effects in other RNABPs of HuR/TTP axis: Influence of TTP knockdown on TIS11 family and HuR RNA binding proteins in human endometrial epithelial (EECC) and endometriotic (12Z) cells. Transcript *ZFP36* which encodes for TTP was significantly lower in both EECC and 12Z cells (Panels A,E). Transcripts for *ZFP36L1* and *ELAVL1* were also downregulated in EECC cells treated with TTP siRNA (panels B,D). The TTP family member *ZFP36L2* did not statistically differ upon knockdown of TTP in EECCs (panel C). On the other hand, transcripts for *ZFP36L2* and *ELAVL1* were upregulated in 12Z cells treated with TTP siRNA (panels G,H). Transcripts for *ZFP36L1* do notstatistically differ when knocked down with TTP siRNA in 12Zs (panel F). Expression data illustrated as mean ± SEM. *P < 0.05. Each group (treated and control) consisted of three wells performed in triplicates. Data is derived from three independent experiments; n = 3.

Knockdown of TTP/*ZFP36* in EECCs (Fig. 5A; p < 0.05) resulted in significant downregulation of *ZFP36L1* as well as *ELAVL1* in treated EECC cells compared to scrambled controls (Fig. 5B,D; p < 0.05). Although not statistically significant, a trend was observed of lower *ZFP36L2* in the treated group compared to controls (Fig. 5C; p = 0.09). On the other hand, knockdown of TTP/*ZFP36* in endometriotic 12Z cells (Fig. 5E; p < 0.05) resulted in significant upregulation of TTP family member *ZFP36L2*, and *ELAVL1* in treated compared to control 12Z cells (Fig. 5G,H; p < 0.05). There was no change in expression for TTP family member, *ZFP36L1* (Fig. 5F).

### Inflammatory cytokine mRNAs are differentially regulated in the absence of TTP/ZFP36

**Figure 6:**
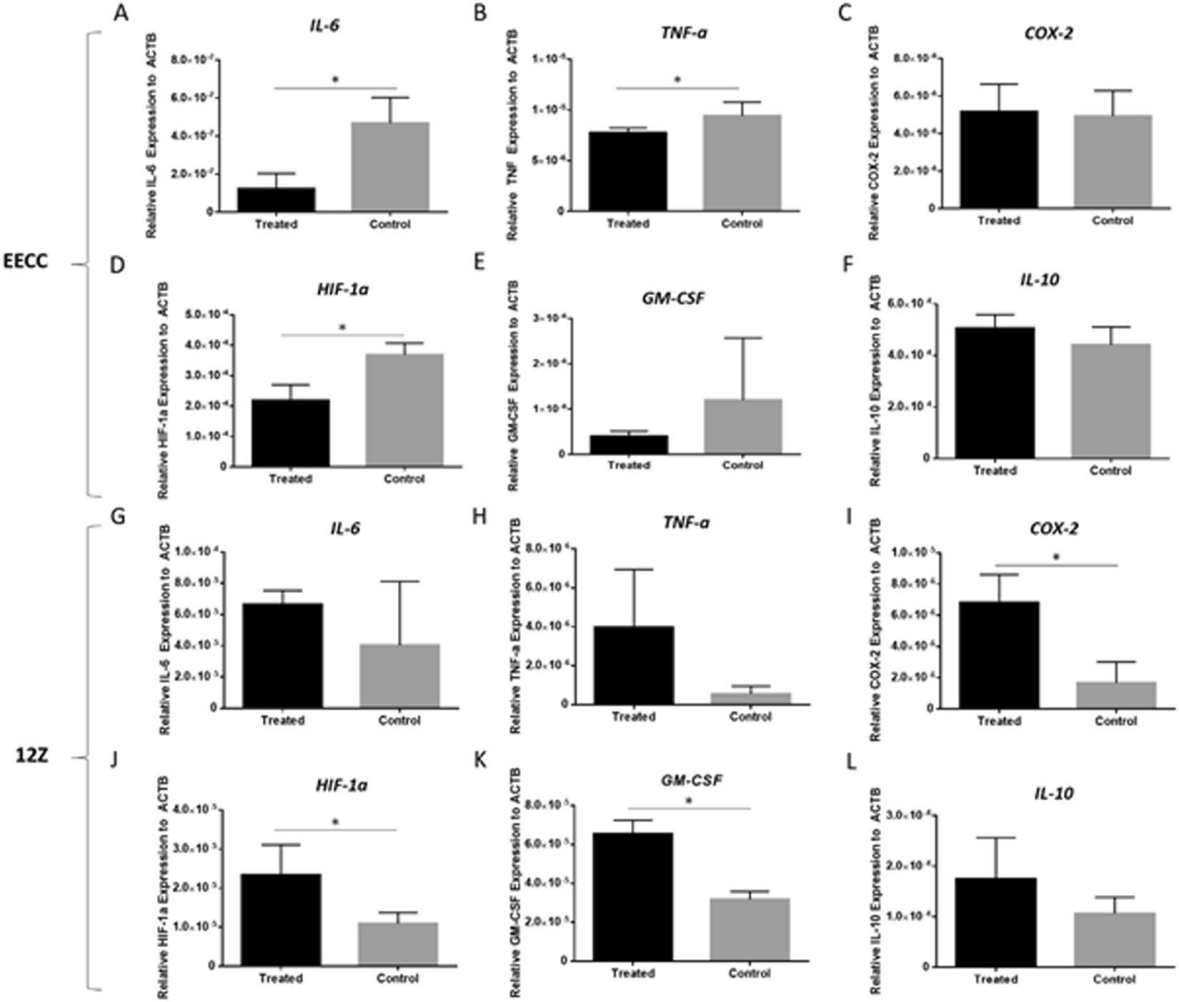
TTP knockdown results in distinct endometriotic (12Z) and endometrial epithelial (EECC)-specific inflammatory profiles: *In vitro* downstream mRNA analysis of inflammatory cytokines associated with RNA binding protein regulation in TTP knockdown EECCs and 12Zs. Transcripts for *IL-6*, *TNF-α*, *HIF-1α* were downregulated in EECC cells treated with TTP siRNA (Panels A,B,D) compared to scrambled controls. Transcripts for *COX-2*, *GM-CSF*, and *IL-10* do not differ in TTP knockdown EECCs (Panels C,E,F). On the other hand, TTP knockdown results in upregulation of *COX-2*, *HIF-1α* and *GM-CSF* in endometriotic epithelial 12Z cells treated with TTP siRNA compared to scrambled controls (Panels I–K). Transcripts for *IL-6*, *TNF-α, and IL-10* do not differ in TTP knockdown 12Zs (Panels G,H,L). Expression data illustrated as mean ± SEM. *P < 0.05. Each group (treated and control) consisted of three wells performed in triplicates. Data is derived from three independent experiments; n = 3.

In order to examine whether TTP interacts with cytokines responsible for inflammation, selected cytokines (GM-CSF, IL-6, IL-10, HIF-1α COX-2, TNF-α) associated with inflammation were examined at mRNA level in TTP knockdown 12Z and EECCs. Pro-inflammatory cytokine *IL-6* was significantly downregulated in TTP knockdown EECCs compared to scrambled control (Fig. 6A; p < 0.05). In a similar manner, the pro-inflammatory cytokine *TNF-a* was also significantly downregulated in TTP knockdown EECCs compared to the scrambled control (Fig. 5B; p < 0.05). The mRNA for *COX-2* (regulator of inflammation) was slightly elevated, although not statistically significant (Fig. 6C). The mRNA encoding for *HIF-1α* was significantly downregulated in TTP knockdown EECCs compared to scrambled control (Fig. 6D; p < 0.05). In 12Z cells, transcripts for *IL-6* and *TNF-α* did not change (Fig. 6G,H) and *COX-2*, *HIF-1α* and *GM-CSF* were significantly upregulated in TTP knockdown cells compared to controls (Fig. 6I,K; p < 0.05).

### Analysis of inflammatory cytokines associated with RNABP regulation in the supernatants of TTP knockdown endometrial epithelial choriocarcinoma cells (EECCs)

**Figure 7:**
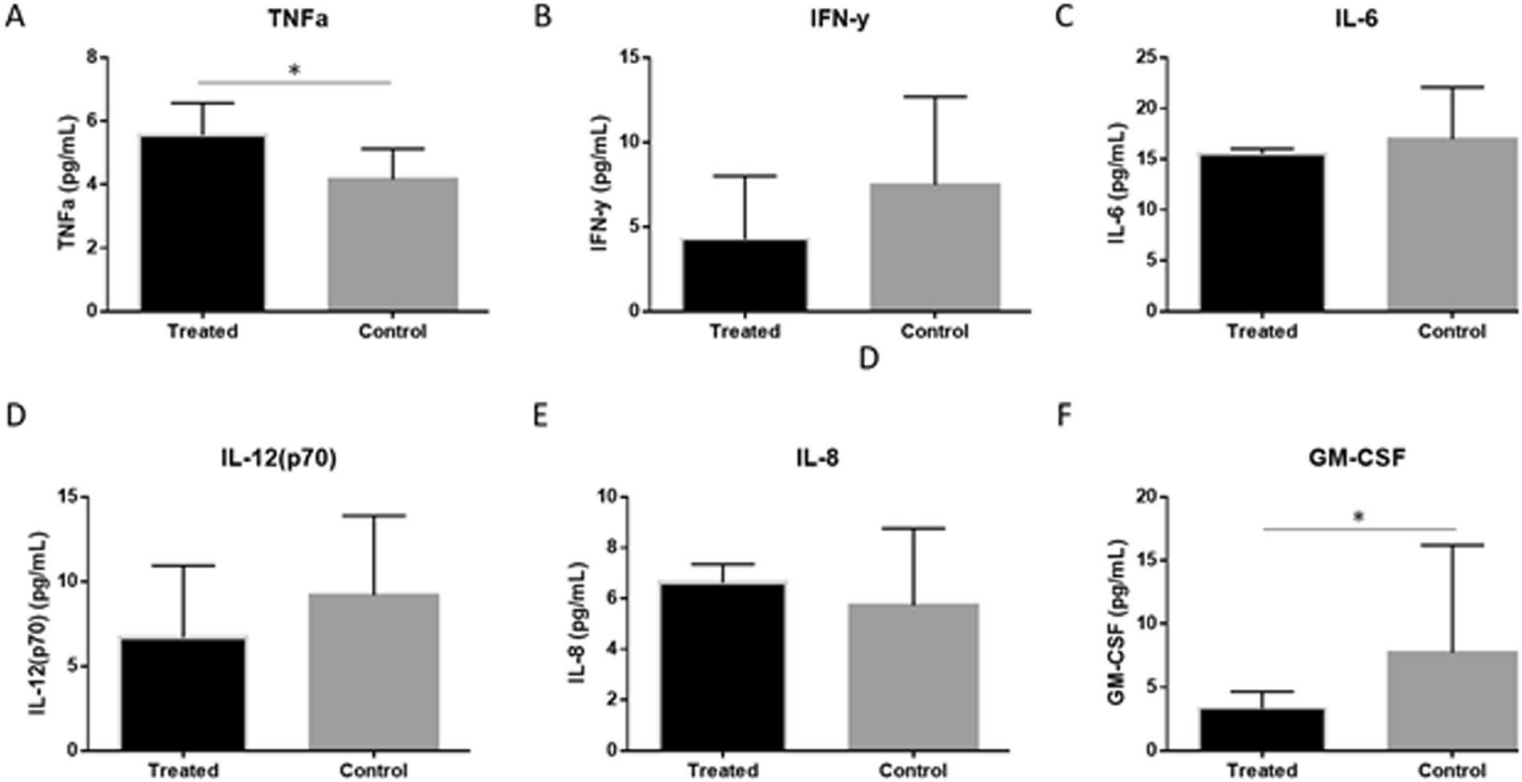
TTP knockdown results in higher levels of TNF-α cytokine: *In vitro* downstream protein analysis of inflammatory cytokines associated with RNA binding protein regulation in TTP knockdown EECCs. Although mRNA transcripts of *TNF-α* was significantly higher in control EECCs compared to TTP knockdown cells, the opposite was observed at the protein level using multiplexing (Panel A). Other inflammatory cytokines for IFN-γ, IL-6, IL-12(p70), IL-8 and GM-CSF did not differ at protein level (Panels B–F). Expression data illustrated as mean ± SEM. *P < 0.05. Data is derived from three independent experiments; n = 3.

Inflammatory cytokine protein profiles (GM-CSF, IFN-γ, IL-1B, IL-2, IL-4, IL-6, IL-10, IL-12(p70), MCP-1, TNF-α) were examined in the supernatants of TTP knockdown EECCs. In contrast to the downregulated mRNA of *TNF-a*, significantly higher TNF-α protein was detected in TTP knockdown compared to scrambled control (Fig. 7A; p < 0.05). Although not statistically significant, a trend was observed of downregulated IFN-γ and GM-CSF in TTP knockdown EECCs compared to controls (Figs 6F and 7B; p = 0.1).

### Localization of HuR and TTP in both ectopic and eutopic endometrium in a mouse model of endometriosis

**Figure 8:**
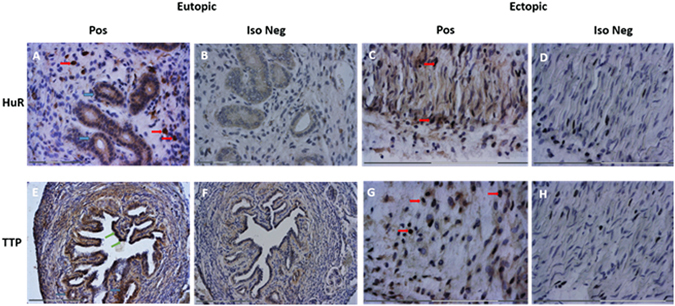
HuR and TTP is immunolocalized throughout the endometrial microenvironment in eutopic and ectopic endometrium from a mouse model of endometriosis. HuR localization patterns were examined in eutopic (Panels A,B) and ectopic (Panels C,D) endometrium. HuR immunostaining was primarily found in glandular epithelium (blue arrows; brown staining prominent in panels A), and endometrial stroma (red arrows, Panels A,C) from both eutopic and ectopic endometrium TTP was localized primarily in glandular epithelium (blue arrows; Panel A), endometrial epithelium and luminal epithelium (green arrows; prominent staining in Panel E compared to isotype negative in Panel F) in eutopic endometrium. High levels of TTP immunolocalization was noted in endometrial stroma in ectopic lesions (Panel G compared to isotype negative Panel H). Images are representative of 5 slides from 6 mice from 2 experiments (n = 3 day 7, n = 3 day 17). Panels A,B original magnification, 100X, scale bar: 75 µm. Panels C,D,G,H original magnification, scale bar: 300 µm 200X, Panels E,F original magnification, 40X, scale bar: 300 µm.

HuR was localized throughout the tissue microarchitecture of both eutopic and ectopic endometrium. Staining was prominent primarily in glandular epithelium, endometrial stroma as well as endometrial epithelium (Fig. 8A and C). HuR immunostaining in the endometrial stroma was primarily cytoplasmic, with low to moderate levels of nuclear staining as well in both eutopic and ectopic endometrium. TTP was localized primarily in glandular epithelium, endometrial epithelium and luminal epithelium (Fig. 8E compared to isotype negative Fig. 8F). High levels of TTP immunolocalization was noted in stromal epithelium in ectopic lesions (Fig. 8G compared to isotype negative Fig. 8H).

## Discussion

In this study we sought to characterize the HuR/TTP axis in endometriosis with a focus on the inflammatory cytokines they have been shown to regulate. We performed initial characterization experiments of HuR/TTP axis in our patient cohorts recruited from both USA and Canada. Our group also sought to understand how a disruption in this axis may influence other RNA binding protein family members as well as their inflammatory targets in both endometriotic and non-endometriotic epithelial cell lines. Based on our previous work with RNA binding proteins in recurrent pregnancy loss^25^, we hypothesized that these RNA binding proteins will be differentially expressed in endometriosis patients.

At the mRNA level, RNA binding proteins from HuR/TTP axis are expressed in lower quantities in endometriosis patients compared to normal endometrium from healthy fertile women (with exception of *ZFP36*). Transcripts for *ZFP36L1* were significantly lower in women with endometriosis in both eutopic endometrium and ectopic endometriotic tissues compared to control fertile women. This finding also translated over for *ELAVL1* and to a lesser extent, *ZFP36L2*, whereby only the eutopic endometrium differed from normal fertile control endometrium. These findings are a stark contrast to our protein findings of the HuR/TTP axis. Although to the best of our knowledge, no endometriosis-specific characterizations of TTP family have been previously performed, these findings are opposite to those observed in some ovarian cancer cell lines, whereby TTP total mRNA transcript copies of the family member *ZFP36L1* were elevated when compared to normal ovarian cell line^20^. This suggests to us that there may be different mechanisms at play in endometriosis influence which RNABPs compared to cancer pathogenesis.

Our patient ELISA protein expression data of both TTP and HuR reveal differential expression between endometriotic lesions and eutopic endometrium from patients compared to control endometrium from healthy fertile women. This suggests that the downregulation of these RNA binding proteins at the site of the lesion as well as in the eutopic endometrium from these patients may in part be linked to the chronic inflammation within the peritoneal microenvironment. Indeed, endometriosis patients have elevated level of pro-inflammatory cytokines in the peritoneal fluid, blood as well as in the ectopic lesions itself suggesting that endometriotic lesions are probably primary drivers of inflammation. We used a different patient cohort that received Dienogest (steroidal progestin of the 19-nortestosterone group), and conducted western blotting to validate our ELISA findings. Despite the fact that these patients were on therapy, we noticed similar downregulation of TTP and HuR in ectopic lesions observed in our patient cohort that did not receive any hormonal therapy for 3 months prior to laparoscopic surgery. The rationale behind including this patient subset was to compare whether the anti-inflammatory effects of Dienogest would have an impact on HuR/TTP axis deficiencies observed in our patients with no hormonal therapy. Previous studies by Karipcin *et al*.^28^ have shown decreased HuR expression in mid-late proliferative and early-mid secretory phases in ectopic lesions from their endometriosis patient cohort. Nevertheless, findings from both of our patient cohorts display dysregulation of HuR/TTP axis, suggesting that the anti-inflammatory effects of Dienogest are independent of the axis^29^. The ratio of mRNA stabilizers/destabilizers may be mediated by the menstrual cycle and different ovarian hormones, and aberrations in this ratio may provide cellular survival advantage to the ectopic endometrial lesion. In comparison to findings by Karipcin *et al*.^28^, further studies will need to be performed to examine HuR/TTP axis throughout the menstrual cycle.

Additionally, our *in vitro* TTP knockdown downstream protein results support this association since TNF-α was found to be elevated in the endometrial epithelial cells and differed at the mRNA level. Importantly, HuR/TTP axis ratio changes are distinctly different in 12Z endometriotic cells compared to non-endometriotic EECC cells. Downregulation of TTP via siRNA in EECCs caused all other RNA binding proteins examined, including HuR, to decrease in expression. Conversely, in TTP downregulated endometriotic 12Z cells, HuR as well as other TIS11 family of RNA binding proteins were upregulated. This endometriosis-specific change in HuR/TTP ratio in the 12Z cells may be due to chronic inflammatory *milieu* these cells originally were exposed and present in them. Indeed, previous findings have pointed to a unique endometrial microenvironment in endometriosis patients that contain signature molecular changes, in part due to chronic inflammation persisting in the peritoneal cavity of these women^9, 30, 31^. Our target inflammatory cytokine profiling in TTP knockdown cells and mRNA findings in these same cells yielded contrasting findings. Inflammatory cytokines *IL-6* and *TNF-α* mRNA was significantly higher in control group compared to TTP knockdown EECCs. The opposite effect has been noted for *IL-6* in embryonic fibroblasts from TTP-deficient mice^32^ as well as *TNF-α* and in TTP-silenced macrophages^33^. This opposite effect was also observed between protein and mRNA for TTP’s major known target, TNF-α and no observed changes were noted for IL-6, which suggests to us that other posttranscriptional regulation may be at play in the endometrium. Additionally, *HIF-1α* transcripts were significantly lower in TTP knockdown EECCs, however *COX-2*, an inducer of HIF-1α does not differ and although not statistically different, is expressed at moderately higher at mRNA levels in the same treated group compared to controls. Recent studies have established links between microRNAs as well as other regulators (lncRNAs, PIWI, etc.) and RNA binding proteins including both TTP and HuR. Specifically, TTP has been shown to promote the microRNA *let-7* via down-regulation of *Lin28* mRNA and down-regulation of *let-7* in serum of mice with endometriosis shows implication of this microRNA in endometriosis^34, 35^. The microRNA *miR-29a* has also been shown to suppress TTP^36^ and our recent data also suggest that *miR-29c* contributes to progesterone resistance in endometriosis^37^. These unique interactions may be contributing to this aberrant change in expression^38^, although we do not discount other forms of regulation (i.e. hormonal) occurring as well.

Tissue-wide profiling experiments examining TTP family mRNA expression have shown moderate to high expression levels of TTP family members in the female reproductive tract, including the ovary^20^. Additionally, HuR has been previously shown to be localized throughout the epithelium of eutopic and ectopic endometriotic lesions, with specific primary localization occurring in the nucleus^28^. We sought to obtain a basic understanding of TTP and HuR localization patterns using our syngeneic mouse model of endometriosis in order to compare and contrast to the aforementioned findings. Indeed, we found similar localization patterns to those previously documented in the normal eutopic endometrium and found that both of these important RNABPs are localized throughout the ectopic endometrial lesions as well. This finding is encouraging and further supports the physiological relevance of these RNABPs in the context of endometriosis and other female reproductive pathologies. Future studies in the mouse model will be useful to determine if modulation of HuR/TTP axis would impact endometriotic lesion growth and survival.

Overall, our findings suggest that the HuR/TTP axis of RNA binding proteins should be examined together as a ratio, rather than individually. In this study, we have shown that aberrations in the ratio of the HuR/TTP axis can cause molecular differences in both endometriotic as well as non-endometriotic endometrial epithelial choriocarcinoma cells. This leads us to believe that aberrations in the ratio of HuR/TTP axis can also have similar molecular changes in the local peritoneal microenvironment. Our *in vitro* TTP knockdown experiments demonstrate that RNABPs are regulated in the absence of ZFP36, although the exact mechanisms have yet to be determined. These studies also point toward the existence of a functional role of TTP/*ZFP36* in endometriotic epithelial 12Z and EECCs. Limitations in our study include limited sample sizes of patients, which did not allow us to further group our patient data sets by stage of disease, although most of these patients were classified as having early stage (I-II) of endometriosis. We also acknowledge the fact that the TTP/*ZFP36* knockdown mechanistic experiments were performed only using an *in vitro* approach. Future studies will aim to recapitulate these findings in an *in vivo* model and to examine which exact mechanisms are influencing the HuR/TTP axis environment in unique subsets of endometriosis patients.

To the best of our knowledge, this work is the first to categorize the HuR/TTP axis in the context of chronic inflammation in endometriosis patients compared to menstrual stage matched fertile controls. We used matched ectopic and eutopic endometrial biopsies to see understand changes in the same patient subset. Overall we demonstrate that: 1) Differential expression of TTP and HuR at the site of the ectopic endometriotic lesion and eutopic endometrium in women, further supported by data from our mouse model of endometriosis. 2) RNA binding proteins are regulated in the absence of TTP/*ZFP36*. 3) Unique endometriosis-specific inflammatory cytokine expression profiles in TTP knockdown endometriotic 12Z and EECCs. Although speculative, our data suggest that the HuR/TTP axis likely works in combination with specific microRNAs and lncRNAs to regulate inflammation both at the site of the ectopic lesion and in the local peritoneal microenvironment. Future work will aim to pinpoint mechanisms of HuR/TTP axis in direct destabilization of inflammatory cytokines at the site of the endometriotic lesions using reporter assays.

## Methods

### Ethics approval

Ethics approval for this study was provided by the Health Sciences Research Ethics Board, Queen’s University, Kingston, Ontario, Canada. Human ectopic endometriosis as well as eutopic endometrial tissue samples were collected from patients with endometriosis as per approved protocols and guidelines. Additionally, endometrial samples were collected from control subjects comprising healthy women after informed consent with the use of a protocol approved by the Institutional Review Committees at Greenville Health Systems, Greenville, South Carolina, and the University of North Carolina, Chapel Hill, North Carolina (IRB protocol number. Pro00000993). All methods were performed as per institutional approved guidelines.

### Sample collection from endometriosis patients and control women

Matched human endometrium (n = 22) and endometriotic lesions (n = 22) from endometriosis patients were provided by Greenville Hospital Systems, Greenville, South Carolina, upon informed consent. Patient samples were further grouped into two groups: early stage I-II (n = 13), and advanced stage III-IV (n = 9). Eutopic endometrium samples were obtained by Pipelle sampling during time of laparoscopic surgery for removal of endometriotic lesions. The patient samples used in this study from Greenville Hospital were comprised of women previously diagnosed with infertility and/or pelvic pain. Both patients and fertile control subjects from Greenville Hospital were free from hormonal therapy for 3 months before the collection of samples. The stage of endometriosis was determined based on the revised American Society of Reproductive Medicine criteria^39^. For control samples, endometrial biopsies (n = 16) were obtained by means of Pipelle sampling from healthy fertile women who underwent tubal ligation at the University of North Carolina. All sixteen healthy control subjects had no signs or symptoms of endometriosis or fertility problems and history of pregnancy. All patients and healthy control subjects were at the secretory phase of the menstrual cycle when samples were obtained. Samples obtained were snap-frozen with the use of liquid nitrogen and then stored at −80 °C until further use. Patient samples from The Ottawa Hospital used for Western blotting studies comprised of matched human endometrium (n = 8) and endometriosis samples (n = 8) from endometriosis patients. These patients samples comprised of women diagnosed with infertility and/or pelvic pain and were receiving Dienogest before collection of samples.

### Mouse model of endometriosis

BALB/cByJ mice (n = 8) (Jackson Laboratory, USA) were housed in cages of 3–4 mice. All animal experiments were performed under Canadian Council of Animal Care guidelines and protocols approved by Queen’s University Institutional Animal Care Committee (Protocol number 2013–061). Donor mice (n = 2) were sacrificed, uterine horns removed, and placed in a Petri dish containing PBS. The endometrium was separated from myometrium, cut into 1 mm^3^ segments, washed in PBS, and kept on ice until surgically implanted in mice. Prior to surgery, recipient mice were anesthetized with 3% isofluorane vaporizer. Small incisions were made in the abdomens of recipient mice and donor mouse endometrium were placed into the left side of the peritoneal cavity of recipient mice using bonding agent. All recipient mice were sacrificed at Days 7 (n = 3) and 17 (n = 3) following surgery. Ectopic lesions were removed and stored in 4% paraformaldehyde prior to tissue processing.

### *In vitro* downregulation of TTP in 12Z and endometrial carcinoma cell lines

Silencing of TTP was performed using a siRNA-based approach in EECC and 12Z cells. In these experiments, EECCs represent generalized epithelial phenotype, while 12Z cells represent epithelium from the endometriotic lesions. Briefly, 200,000 cells were plated in 6 well plates (Sarstedt AG, Germany) in antibiotic-free Dulbecco’s medium (F-10 EECCs, F12 12Z) supplemented with 10% fetal bovine serum (FBS; Sigma-Aldrich, Canada). The 12Z cells were grown as per previously published protocol^40^. Briefly, 12Z cells had addition of 1% sodium pyruvate in the DMEM media and were previously checked for absence of mycoplasma. Cells were grown for 18 hours until 60% confluence and transfected with 1 µg of TTP duplex siRNA (sc-36760, Santa Cruz Biotechnology, Texas, USA) mixed with 8 ul of siRNA transfection reagent and 200 ul siRNA transfection medium (sc-29528 & sc-36868, Santa Cruz Biotechnology, Texas, USA). Scrambled control siRNA (sc-37007, Santa Cruz Biotechnology, Texas, USA) were added to control wells. Cells were incubated for 8 hours at 37 °C and DMEM supplemented with 20% FBS was added and incubated for an additional 24 hours. Medium was aspirated followed by supernatant collection and all cells were collected for downstream applications (RNA and protein extraction). Knockdown efficiency of TTP was determined using qPCR and western blotting approaches and knockdown efficiency cut-off threshold was set at 70%.

### RNA binding protein mRNA detection using quantitative real-time PCR

The mRNA of TTP family as well as the mRNA stabilizer, *ELAVL1* were assessed using quantitative real-time PCR (qPCR). Total RNA from endometriosis, eutopic endometrium patient and control samples as well as cell lysates from 12Z and EECC cultures were reverse transcribed using Superscript II reverse transcription kit (Life Technologies, Canada) as per the manufacturer’s protocol. Primers (Table 1) were designed using Primer3 software (http://frodo.wi.mit.edu/primer3/) from murine and human sequences available on NCBI’s Nucleotide. Real-time PCR was performed using plate-based LC-480 (Roche Diagnostics., Montreal, Canada). Experimental set-up was according to the MIQE guidelines^41^. Relative quantification was performed using *ACTB* as a control gene. Pooled human endometrial and murine cDNA was used as calibrators. Expression of *ACTB* did not differ across groups by one-way ANOVA. All samples were run in triplicates. The run protocol for all genes of interest used was the following: Denaturation: 95 °C, 15 min; Amplification: 45 cycles: 95 °C for 15 s, 55 °C for 30 s, 70 °C for 30 s; Melting Curve: 70–95 °C, at a rate of 0.1 °C per second. Data was analyzed using the ∆∆Ct method.

**Table 1:** mRNAs Assessed by Real-Time PCR.

Gene Name	Primer	Product Size (bp)	GenBank Accession Number
*mZfp36*	for: 5′-CTCCTGCCGAAGGTCTACTA-3′	155	NM_011756
	rev: 5′-TGCCTCAAAGACAGGTGAGTC-3′		
*mZfp36l1*	for: 5′-CAAGGGTAACAAGATGCTCAACTAC-3′	222	NM_007564
	rev: 5′-GAGAAAGAGCGGTCTCGAAAG-3′		
*mZfp36l2*	for: 5′-GCTGCCACCTCCCTAAACTA-3′	190	NM_001001806
	rev: 5′-GCAATGAGCCCGTTATCA-3′		
*mElavl1*	for: 5′-GGCTGGTGCATCTTCATCTAC-3′	176	NM_010485
	rev: 5′-GCCATTGCAGCTTCTTCATAGT-3′		
*hZFP36*	for: 5′-CATGGATCTGACTGCCATCTA-3′	279	NM_003407.3
	rev: 5′-GAAGTGGGTGAGGGTGACAG-3′		
*hZFP36L1*	for: 5′-TCTGCCACCATCTTCGACTT-3′	109	BT019468.1
	rev: 5′-TGCCCACTGCCTTTCTGT-3′		
*hZFP36L2*	for: 5′-ACTCCGATGAGTTTGGGACTT-3′	251	NM_006887.4
	rev: 5′-ATGGGCTGAGGGCTAACTATC-3′		
*hELAVL1*	for: 5′-GTTCAGCAGCATTGGTGAAGT-3′	253	BT009793.1
	rev: 5′-TTCTACGTCCTTCTGGGTCAT-3′		
*hCOX-2*	for: 5′-ATGTTCCACCCGCAGTACA-3′	202	NM_000963.3
	rev: 5′-TTCTACCAGAAGGGCAGGATAC-3′		
*hIL-6*	for: 5′-CCAGAGCTGTCCAGATGAGTA-3′	206	M14584.1
	rev: 5′-TGAGGTGCCCATGCTACA-3′		
*hTNF-α*	for: 5′-CCAGTCTGGGCAGGTCTACT-3′	205	M26331.1
	rev: 5′-GGTTTCGAAGTGGTGGTCTT-3′		
*hGM-CSF*	for: 5′-TGAAGGACTTTCTGCTTGTCAT-3′	225	NM_000758.3
	rev: 5′-TCTTCTGCCATGCCTGTATC-3′		
*hIL-10*	for: 5′-GGCACCCAGTCTGAGAACA-3′ rev: 5′-GCATCACCTCCTCCAGGTAAA-3′	220	M57627.1
*hHIF-1α*	for: 5′-CCATTCCTCACCCATCAAATA-3′	254	AB733094.1
	rev: 5′-TTTGGCAAGCATCCTGTACT-3′		
*mActb*	for: 5′-GGCTGTGCTGTCCCTGTATG-3′	272	NM_007393.5
	rev: 5′-CCATCTCCTGCTCGAAGTCTA-3′		
*hACTB*	for: 5′-GAGGCCTGGACTCTCAACTG-3′	187	AY582799.1
	rev: 5′-AATGAATGGGGGTTGAATGA-3′		

### TTP and HuR ELISA Assay

Total protein was extracted from eutopic (n = 22) and ectopic endometrium (n = 22). Approximately 20 mg of tissue placed in 1.5 mL microcentrifuge tube containing 0.3 g/mL of protease inhibitor cocktail (Sigma-Aldrich, Canada), in 200 µL of PBS. Upon tissue homogenization, samples were centrifuged at 4 °C and supernatant was collected. Protein concentrations were determined using a bicinchoninic acid (BCA) assay (Thermo-Fisher Scientific, Canada) as per kit manufacturer’s instructions. Eutopic and ectopic endometrium protein obtained from endometriosis patients were thawed at room temperature. TTP and HuR were quantified using TTP and HuR immunoassay kits following manufacturer’s protocol (Cat #MBS2602738 and MBS919324, MyBioSource Inc., CA, USA). Briefly, samples were added to 96-well immunoassay plates pre-coated with antihuman TTP and HuR antibodies and following incubation, absorbance was read at 450 nm within 15 mins using SpectraMAX Plus spectrophotometer (Molecular Devices, USA). Sensitivity for TTP and HuR immunoassays were 60.00 and 6.25 pg/ml, respectively.

### Western Blotting

TTP and HuR protein expression in ectopic compared to eutopic endometrium from endometriosis patients from Ottawa Hospital cohort was determined using western blotting. Total protein was extracted from eutopic (n = 8) and ectopic endometrium (n = 8). Approximately 20 mg of tissue placed in 1.5 mL microcentrifuge tube containing 0.3 g/mL of protease inhibitor cocktail (Sigma-Aldrich, Canada), in 200 µL of PBS. Upon tissue homogenization, samples were centrifuged at 4 °C and supernatant was collected. Protein concentrations were determined using a bicinchoninic acid (BCA) assay (Thermo-Fisher Scientific, Canada) as per kit manufacturer’s instructions. All samples were normalized to a protein concentration of 10 µg/µL using PBS and stored at −80 °C. Samples were denatured at 99.9 °C for 5 minutes in a thermal cycler (Bio-Rad, CA, USA). 5 ul of loading dye was pipetted to appropriate wells of 12% Tris-glycine pre-cast gels (Bio-Rad, CA, USA) (12 wells/20 µL) and separated at 120 V for approximately 1 h. Transferring was performed onto PVDF membranes (Thermo-Fisher Scientific, Canada) and run at 100 V for 2 h. Membranes were blocked in 5% skim milk TBS-T solution overnight. 1 µg/mL of Rabbit polyclonal anti-TTP/ZFP36 (Ab83579, Abcam, CA, USA) and 1.0 µg/mL Rabbit monoclonal anti-HuR/ELAVL1 (Abcam Ab200342) antibodies were added as primary antibodies. Membranes were rinsed 3 X at 10 mins with TBS-T solution and 5 X at 5 mins with TBS solution. HRP conjugated goat anti-rabbit IgG 1:2000 (R&D, HAF008), secondary antibody was added in 5% skim milk TBS-T solution to each of the membranes and incubated on a rocker at room temperature (RT) for 2 h. Enhanced chemiluminescence detection was completed with Clarity chemiluminescent substrate solution (Abcam, CA, USA) and imaged on a Kodak X-ray film processor. Membranes were stripped and re-probed for ACTB using 1.5 µg/mL of anti-ACTB mouse monoclonal antibody (Ab20272, Abcam, CA, USA) diluted with 5% skim milk TBS-T solution, and incubated on an electric plate-rocker in a 4 °C refrigerator for 12 h. All films were scanned on a flatbed scanner at 600dpi greyscale and images were analyzed using ImageJ software (NIH, Bethesda, MD) to obtain densitometry values.

### Multiplex cytokine analysis

A 12-plex high sensitivity commercial available multiplex assay was conducted from Eve Technologies, Calgary, Alberta using Luminex xMAP laser bead platform (Bio-Rad, CA, USA). This multiplex assay was customized to cytokines involved in inflammation/inflammatory pathways, and included: GM-CSF, IFNγ, IL-1B, IL-4, IL-5, IL-6, IL-8, IL-10, IL-12p70, IL-17, IL-23, TNF-α. Briefly, color-coded polystyrene beads were coupled with capture antibodies for each cytokine. A total of 50 µl of each protein samples were aliquoted and normalized. The normalized protein samples were incubated in a mixture containing the capture antibodies conjugated to magnetic beads in a custom 96-well plate. The plate was incubated in a dark room on a shaker at room temperature for 30 mins. The plate was washed three times with wash buffer. Detection antibody (25 μL) was added with antibody diluent and incubated in the dark on a shaker at RT for 30 mins. Upon rinsing three times with wash buffer, 50 μL streptavidin-phycoerythrin fluorescent conjugate was added to all wells. The plate was incubated for 10 mins and washed and loaded. The standards for this assay were provided by the manufacturer as a lyophilized cocktail of proteins and run for each individual target analyte. Standard curve dilutions were run on the assay. Increasing fluorescent intensity signals were read as fluorescence intensity (FI) values, which correspond to protein bound to each analyte bead. The observed concentration (OC) for each target analyte was calculated against standard curve regression.

### Immunohistochemical localization of TTP and HuR in endometriotic lesions from mouse model

Ectopic endometriotic lesion (n = 3) and eutopic endometrium (n = 3) were embedded in paraffin and cut (5 µm) and mounted on charged slides (Superfrost, Thermoscientific, Canada). Paraffin-embedded endometrium were used for TTP and HuR immunolocalization experiments using previously published IHC protocol^25^. Briefly, antigen retrieval was performed with citric acid buffer boiling for 2 mins. Blocking was performed on all sections using 1% bovine serum albumin (BSA) for 1.5 hours at room temperature. Rabbit polyclonal to tristetraprolin (sc-12563, Santa Cruz Biotechnology, USA) and Rabbit monoclonal anti-HuR (ab200342, Abcam plc., UK) were added at 1.5 µg/mL and 0.8 µg/mL, respectively. Biotin-conjugated secondary antibody was used (K0690, DakoCytomation, USA) was added to all sections and incubated for 45 mins at room temperature. DAB was utilized as chromogen and was controlled for 10 seconds and 15 seconds per section for TTP and HuR, respectively. Counterstaining was done using haematoxylin for 15 seconds and upon rinsing, slides were dehydrated and coverslips were added. All slide images were taken using a ZEISS observer microscope and analyzed using ZEISS imaging software (Zeiss, Canada).

### Statistical Analysis

*In vivo* patient mRNA and protein data were analyzed by one-way analysis of variance (ANOVA). Patient outliers were removed using ROUT outlier removal from Graphpad Prism 6.05 software (Q = 5%). Mouse model data were analyzed using parametric paired *t-*test. The *in vitro* silencing experiments used an unpaired Student’s *t-*test. A p < 0.05 was considered as statistically significant.
